# Fast Quantum Rabi Model with Trapped Ions

**DOI:** 10.1038/srep38961

**Published:** 2016-12-12

**Authors:** Héctor M. Moya-Cessa

**Affiliations:** 1 Instituto Nacional de Astrofísica, Óptica y Electrónica, Calle Luis Enrique Erro 1, Santa María Tonantzintla, Puebla, 72840 Mexico

## Abstract

We show how to produce a fast quantum Rabi model with trapped ions. Its importance resides not only in the acceleration of the phenomena that may be achieved with these systems, from quantum gates to the generation of nonclassical states of the vibrational motion of the ion, but also in reducing unwanted effects such as the decay of coherences that may appear in such systems.

Trapped ions are considered one of the best candidates to perform quantum information processing. By interacting them with laser beams they are easy to manipulate, because of the fact that they can be individually addressed, which makes them an excellent choice for the production of nonclassical states of their vibrational motion.

The trapping of individual ions also offers many possibilities in spectroscopy^1^, in the research of frequency standards^2^,^3^, in the study of quantum jumps^4^, to name some applications. To make the ions more stable in the trap, increasing the time of confinement, and also to avoid undesirable random motions, it is needed that the ion be in its vibrational ground state which may be accomplished by means of an adequate use of lasers^5^,^6^.

Because of the high nonlinearities of the ion-laser interaction its theoretical treatment is a nontrivial problem^7^,^8^,^9^,^10^,^11^,^12^,^13^. Even in the simplest cases of interaction one has to employ physically motivated approximations in order to find a solution. A well-known example is the *Lamb*-*Dicke* approximation, in which the ion is considered to be confined within a region much smaller than the laser wavelength. Other examples are optical and vibrational rotating wave approximations that are usually performed in order to find simpler Hamiltonians. In fact, perturbative methods have been used to obtain better analytical approximations^14^.

Recently it was shown that the quantum Rabi model^15^ could be engineered via the interaction of two laser beams with a trapped ion^16^. Pedernales *et al*. did it by slightly detuning both laser beams from the blue and red side bands, allowing them to construct a Hamiltonian of the Rabi type and reaching all the possible regimes. However, because the parameters involved are much smaller than the vibrational frequency of the ion, *ν*, the ion can suffer losses that lead to the decay of Rabi oscillations^17^,^18^. There have been attempts to explain such loss of coherences via laser intensity and phase fluctuations^19^. Also recently, Puebla *et al*.^20^ have shown how to produce, in a robust manner, a quantum Rabi model, in a variety of parameter regimes, by manipulating ions with laser beams.

We will show here two approaches in which we can engineer a fast Quantum Rabi model (QRM), fast in the sense that the parameters involved in the interaction may be of the order of *ν*. Instead of two off-resonant lasers^16^, we use only one resonant beam.

## Ion-laser interaction

We can write the Hamiltonian of the trapped ion as


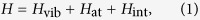


where *H*_vib_ is the ion’s center of mass vibrational energy, *H*_at_ is the ion internal energy, and *H*_int_ is the interaction energy between the ion and the laser. The vibrational motion can be approximated by a harmonic oscillator. Internally, the ion will be modelled by a two level system. In the interaction between the ion and the laser beam, we will make the dipolar approximation, so we will write the interaction energy as 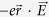, where 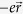 is the dipolar momentum of the ion and 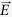 is the electric field of the laser, that will be considered a plane wave. Thus, we write the Hamiltonian, after an optical rotating wave approximations as


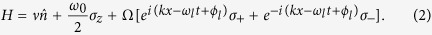


The first term in the Hamiltonian is the ion vibrational energy; in the ion vibrational energy, the operator 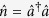 is the number operator, and the ladder operators 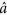 and 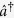 are given by the expressions


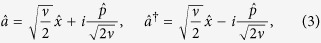


where we have set the ion mass equal to one. Also, for simplicity, we have displaced the vibrational Hamiltonian by *ν*/2, the vacuum energy, that without loss of generality may be disregarded.

The second term in the Hamiltonian corresponds to the ion internal energy; the matrices *σ*_*z*_, *σ*_+_, and *σ*_−_ are the Pauli matrices, and obey the commutation relations


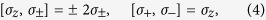


and *ω*_0_ is the transition frequency between the ground state and the excited state of the ion.

By considering the resonant condition, *ω*_0_ = *ω*_*l*_, and transforming to a picture rotating at *ω*_*l*_ we obtain the Hamiltonian


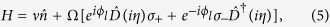


where we have defined the so-called Lamb-Dicke parameter


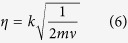


that is a measure of the amplitude of the oscillations of the ion with respect to the wavelength of the laser field represented by its wave vector *k*.

If we consider the condition 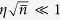, where 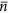 is the average number of vibrational quanta, we can expand the Glauber displacement operator^21^ in Taylor series^22^,^23^)


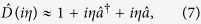


such that the Hamiltonian (2) reads


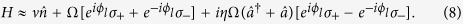


By setting *ϕ*_*l*_ = *π* and making now a rotation around the *Y* axis (by means of the transformation 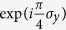), with *σ*_*y*_ = *iσ*_−_ − *σ*_+_, we obtain the usual form of the Rabi Hamiltonian


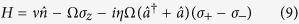


If we take now *ν* = −2Ω, and we use the rotating wave approximation, the Hamiltonian reduces to the anti-Jaynes-Cummings (AJC) interaction Hamiltonian


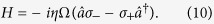


On the other hand, if we set *ϕ*_*l*_ = 0 and follow the same procedure we obtain


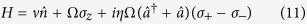


that, by taking *ν* = 2Ω, and using the rotating wave approximation now reduces to the Jaynes-Cummings (JC) interaction Hamiltonian


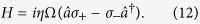


Because the transformation to achieve the quantum Rabi model from the ion-laser interaction was a simple rotation, observables pertaining the vibrational motion of the ion are left unchanged. However measurements on atomic states result in other atomic properties, namely the atomic inversion (〈*σ*_*z*_〉) gives information about the dipole moment (〈*σ*_*x*_〉) and viceversa.

Up to here we have been able to construct the Rabi interaction with a set of parameters that do not allow all the regimes because 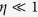 only permits the JC and AJC interactions. However, we should stress that this is a much faster interaction than the one produced by Pedernales *et al*.^16^ as Ω is the order of *ν*.

## Fast Quantum Rabi Hamiltonian

We turn out attention again to the Hamiltonian given in equation (5) and set *ϕ*_*l*_ = 0


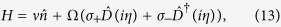


we rewrite Equation 13 in a notation where operators acting on the internal ionic levels are represented explicitly in terms of their matrix elements, as


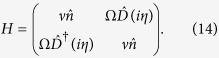


We follow the unitary operator procedure introduced by Moya-Cessa *et al*.^23^,^24^ and define the transformation matrix (whose elements are displacement operators)


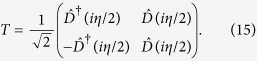


It is possible to check after some algebra that


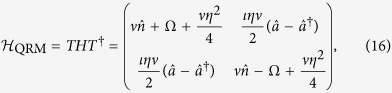


that, after returning to the former (spin matrices) notation reads


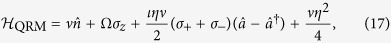


that is nothing but the quantum Rabi Hamiltonian plus a constant term that can be disregarded.

The speed of the the quantum Rabi model is governed by the Rabi frequency^15^, Ω, and may be improved simply by increasing its value. For instance, in order to produce the vibrational wave approximation, it is asked that 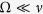, which in experiments with ^40^Ca^+ ^25,26 have been produced: *ν* = 2*π* × 1.36 MHz while the Rabi frequency is of the order of KHz. In the above equation, Ω has no restriction and may be of the order of *ν* increasing the speed about three orders of magnitude.

Because Hamiltonians (14) and (17) are connected by a unitary (similarity) transformation, they are equivalent and any solution found for one of them, by means of the transformation (15) delivers a solution to the other.

A solution for the Rabi model has been given recently by Braak^27^, and therefore this can be extended to the ion-laser interaction, for instance, we can write the evolution operator for the ion-laser interaction as


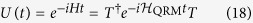


and we can further choose different initial conditions for the ion’s internal and external degrees of freedom in order to have the solution applied to simpler transformed states. For instance, if we consider the initial entangled state


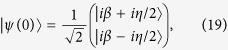


the solution to the complete ion-laser interaction Hamiltonian reads


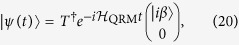


*i*.*e*., simply the solution to the quantum Rabi model for a coherent state for the field and an excited state for the atom.

Otherwise, if instead we use the initial state


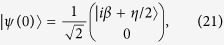


the solution now reads


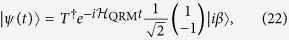


which means that the evolution operator for the quantum Rabi model has to be applied to an initial condition for the field in a coherent state and the atom in a pure superposition of its ground and excited states.

We should stress that if we had considered a detuning, an extra term would have to be added to Equation 17 that would represent an extra static electric field interacting with an atomic dipole^28^.

Also note that in the above Hamiltonian we have not made any assumptions on the parameters Ω and *η*.

The transformation (15) has already been used to find (families of) exact solutions to the QRM^28^. Taking advantage of the fact that 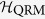 is similar to *H*, we can obtain families of eigenstates of the quantum Rabi Hamiltonian, for instance, the lowest (unnormalized) eigenstate found for the ion-laser interaction


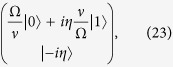


with eigenvalue *ν*, translates to an eigenstate of the quantum Rabi Hamiltonian as


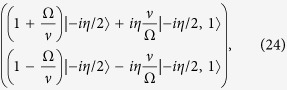


where 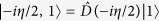 is a so-called displaced number state^29^,^30^.

This correspondence is very useful, since it enables one to map interesting properties of each model onto their counterparts in the other. For instance ways of realizing substantially faster logic gates for quantum information processing in a linear ion chain^31^, their stability conditions^32^,^33^ and gates not only insensitive to the temperature of the ions, *i*.*e*., independent of the initial motional state, but that also work outside the Lamb-Dicke regime^34^.

## Effective Hamiltonian

Now we show how to produce a fast dispersive Hamiltonian. Pedernales *et al*.^16^ showed that it is possible to build such a Hamiltonian by using two slightly of resonant laser beams tuned almost to the blue and red sidebands. However, as the parameters they used are in general much smaller than *ν*, the dispersive interaction constant, may be very small. Here, we take advantage of the fact that the Hamiltonian given in (17) has not been approximated and therefore there are no restriction on the values of their parameters. By transforming the Hamiltonian (17) with the unitary operators^35^


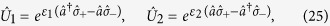


with ε_1_, 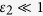,


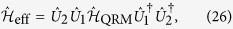


and setting


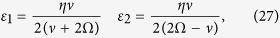


remaining up to first order in the expansion 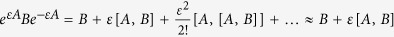, *i*.*e*., doing a small rotation^35^, we obtain the so-called dispersive Hamiltonian


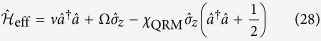


where the effective interaction constant has the form


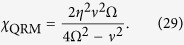


## Discussion

Note that most regimes may be achieved with this *fast* treatment: Jaynes-Cummings and anti Jaynes-Cummings were produced with the first method 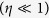 and may be produced with the last method, where the following regimes may also be achieved

- decoupling regime may also be achieved 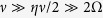
- the two-fold dispersive regime, where *ην*/2 < *ν*, 2Ω, |2Ω − *ν*|, 2Ω + *ν*|,
- the deep and ultra strong coupling regime, for *η* > 2.

The last regimes may be achieved, as in principle, one can produce large Lamb-Dicke parameters^9^,^11^,^36^,^37^. It should again be stressed that, because decay actually happens in ion-laser interactions^17^,^19^ it is of great importance to have fast interactions^31^ in order to minimise such unwanted phenomena that avoids the generation of nonclassical states, quantum gates, and other important quantum effects.

Finally, a drawback of this approach is that, because the frequency of the trap can not be taken to zero, simulation of the Dirac equation can not be considered. Another would be that some properties of the model can not be explored because parameters can not be tuned during simulation time.

## Additional Information

**How to cite this article**: Moya-Cessa, H. M. Fast Quantum Rabi Model with Trapped Ions. *Sci. Rep.* **6**, 38961; doi: 10.1038/srep38961 (2016).

**Publisher's note:** Springer Nature remains neutral with regard to jurisdictional claims in published maps and institutional affiliations.
